# COVID-19 preventive measures coincided with a marked decline in other infectious diseases in Denmark, spring 2020

**DOI:** 10.1017/S0950268822001145

**Published:** 2022-07-28

**Authors:** Rikke Thoft Nielsen, Tine Dalby, Hanne-Dorthe Emborg, Anders Rhod Larsen, Andreas Petersen, Mia Torpdahl, Steen Hoffmann, Lasse Skafte Vestergaard, Palle Valentiner-Branth

**Affiliations:** 1 Department of Infectious Disease Epidemiology & Prevention, Statens Serum Institut, Copenhagen, Denmark; 2 Department of Bacteria, Parasites & Fungi, Statens Serum Institut, Copenhagen, Denmark

**Keywords:** Bacterial infections, COVID-19, epidemiology, virus infection

## Abstract

We aimed to descriptively analyse the possible impact of the national COVID-19 interventions on the incidence of common infectious diseases in Denmark during spring and summer 2020. This observational study focused on national register data on infections caused by 16 different bacterial and viral pathogens. We included new cases registered between 1 January 2016 and 31 July 2020. The weekly number of new cases were analysed with respect to the COVID-19-related interventions introduced during 2020. We found a marked decrease in infections associated with droplet transmission coinciding with the COVID-19 interventions in spring and summer 2020. These included decreases in both viral and bacterial airway infections and also decreases in invasive infections caused by *Streptococcus pneumoniae*, *Haemophilus influenzae* and *Neisseria meningitidis*. There was also a reduction in cases associated with foodborne transmission during the COVID-19 lockdown period. We found no effect of the lockdown on infections by invasive beta-haemolytic streptococci group B, C and G, *Staphylococcus aureus* bacteraemia, *Neisseria gonorrhoeae* or *Clostridioides difficile*. In conclusion, we found that the widespread interventions such as physical distancing, less travel, hygiene measures and lockdown of schools, restaurants and workplaces together coincided with a marked decline in respiratory infections and, to a smaller extent, some foodborne-transmitted infections.

## Introduction

The outbreak of SARS-CoV-2 started in December 2019 in Hubei, China [1] but quickly spread to the rest of the world. By March 2020, Europe was the new epicentre of the pandemic [2]. This resulted in the implementation of a wide range of preventive measures and restrictions and even society lockdowns across Europe and many other countries worldwide to halt the transmission of SARS-CoV-2. In Denmark, the first COVID-19 case was detected on 27 February 2020 [3]. The main interventions against COVID-19 in Denmark were launched on 12 March 2020 and a gradual relaxation of these restrictions and re-opening of the society was initiated on 15 April 2020 [4].

A few studies have reported the impact of the interventions against COVID-19 on the occurrence of other infectious agents, primarily with a focus on paediatric infections. Vierucci *et al*. found a decrease of infectious diseases in a paediatric unit in Tuscany, Italy during the lockdown [5]. Similarly, Angoulvant *et al*. observed a significant decrease in infectious diseases disseminated through airborne and faecal–oral transmission during the lockdown in paediatric emergency departments in France [6] and Kruizinga *et al*. reported more than a 50% reduction in visits and admissions to paediatric emergency departments during this time [7]. Emborg *et al*. reported a sharp decrease in influenza in Denmark, Norway and Sweden during the 2019–20 influenza season following the interventions against COVID-19 [8] while Folkehelseinstituttet also reported an ongoing reduction in contagious diseases during the COVID-19 pandemic in Norway [9]. A recent study of invasive infections with *Streptococcus pneumoniae*, *Haemophilus influenzae* or *Neisseria meningitidis* in 26 countries also reported a marked decline [10], and a Scandinavian study on *Chlamydia trachomatis* and *Neisseria gonorrhoeae* showed a limited effect on the spread of these two infections [11].

In Denmark, there are several national systems in place for the surveillance of infectious diseases. All results from microbiological analyses from the regional clinical microbiology laboratories are registered in real-time in the Danish Microbiology Database (MiBa) [12]. Consequently, MiBa allows for a universal surveillance of infections in Denmark, and makes it possible to monitor the occurrence of various infectious diseases in real time. Each test result is registered along with sex, age and the unique personal identification number of the patients. By use of the Danish Civil Registration system [13], information on home address for each patient can be retrieved, and regional differences in incidences can therefore be evaluated. For some pathogens, the total number of diagnostic tests can be found in MiBa – for others it is not, as yet, possible.

For some infectious diseases, there is a mandatory laboratory notification by use of data from MiBa. For a number of bacterial pathogens, the surveillance is based on mandatory submission of isolates to the national reference laboratories at Statens Serum Institut (SSI). For other pathogens, surveillance is not mandatory, but is nevertheless conducted either through data from MiBa or through submission of bacterial isolates to the reference laboratories. Such collaborations between SSI and the regional laboratories of clinical microbiology have been conducted through decades and analysis of the received isolates provides valuable information on phenotypes and/or genotypes (e.g. serotype, serogroup).

The aim of this study was to use national microbiological data to examine whether there was an impact of the national COVID-19 interventions during spring and summer 2020 on the occurrence of 16 selected bacterial and viral infectious diseases.

## Methods

In this observational study, we investigated infections with 16 different pathogens with different routes of transmission. Airway infections with droplet transmission were represented by *Mycoplasma pneumoniae*, respiratory syncytial virus (RSV), rhinovirus, metapneumovirus, parainfluenza virus and *Bordetella pertussis* while invasive infections related to airway infections and/or throat colonisation were represented by *S. pneumoniae*, *H. influenzae* and *N. meningitidis*. Infections characterised by foodborne transmission were represented by *Campylobacter* spp. and *Salmonella* spp., and infections characterised by sexual transmission were represented by *N. gonorrhoeae*. For the purpose of comparison, we investigated the incidence of *Clostridioides difficile*, methicillin-resistant *Staphylococcus aureus* (MRSA) cases, *S. aureus* bacteraemia and invasive infections caused by beta-haemolytic streptococci (BHS).

Information on cases came either from MiBa, from the national reference laboratories or a combination of the two (Table 1). Cases are reported as episodes, i.e. each patient–infectious agent combination is only recorded once in any pathogen-specific period (Table 1). Cases between 1 January 2016 and 31 July 2020 were included in this study except for *C. difficile* where we only included confirmed cases from 1 January 2018, due to a new and more complete method of registering cases introduced in 2017. Invasive infections include pathogens detected by culture or PCR from normally sterile sites. For RSV, parainfluenza virus, metapneumovirus, rhinovirus and *M. pneumoniae*, information about the total number of samples analysed as well as the number of positive tests was available, and a positive percentage for the tests could therefore be calculated. For *B. pertussis*, the development in the number of diagnostic samples and percentage of positive tests has briefly been described in [14].

**Table 1.** Overview of case definitions used for different pathogens in the national surveillance system

Pathogen	Case definition	Data source and system of surveillance
*Mycoplasma pneumoniae*, respiratory syncytial virus, rhinovirus, metapneumovirus, parainfluenza virus	First positive test within a season per individual	MiBa, all national cases. Number of diagnostic tests is available
*Bordetella pertussis*	First positive test within a year per individual	MiBa, all national cases. Number of diagnostic tests is available
Invasive *Streptococcus pneumoniae*	First isolate within 30 days per individual	Mandatory submission of isolates. The reference laboratory's isolate databases supplemented by MiBa to cover all national cases
Invasive *Haemophilus influenzae*	First isolate within 30 days per individual	Mandatory submission for Hib, non-mandatory submission for other serotypes. The reference laboratory's isolate databases supplemented by MiBa to cover all national cases
Invasive *Neisseria meningitidis*	First isolate or positive PCR test within 30 days per individual	Non-mandatory submission of isolates at a high coverage. The reference laboratory's isolate database
Invasive beta-haemolytic streptococci	First isolate within 30 days per individual	Non-mandatory submission of isolates at a high coverage The reference laboratory's isolate database
*Campylobacter* spp., *Salmonella* spp.	First positive test within 6 months per individual	All national cases from MiBa are collected in the Register of Enteric Pathogens maintained by SSI and updated with results from the reference laboratory's isolate database (non-mandatory submission)
*Clostridioides difficile*	First positive test within 8 weeks per individual	All national cases from MiBa are collected in the Register of Enteric Pathogens maintained by SSI and updated with results from the reference laboratory's isolate database (non-mandatory submission)
*Neisseria gonorrhoeae*	First isolate or positive PCR test within 21 days per individual	The reference laboratory's isolate database (non-mandatory submission at a high coverage)
*Staphylococcus aureus* from bacteraemia	First isolate within 30 days per individual	The reference laboratory's isolate database (non-mandatory submission at a high coverage)
Methicillin-resistant *Staphylococcus aureus*	First isolate within a year per individual	The reference laboratory's isolate database (non-mandatory submission at a high coverage)

### COVID-19 preventive measures

**Fig. 1.**
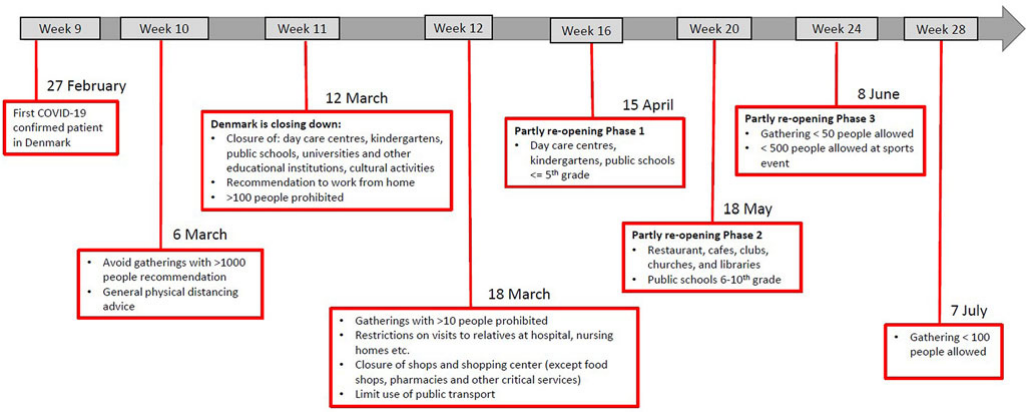
A schematic overview of the COVID-19 interventions implemented in Denmark during the weeks 9–28, 2020.

Denmark introduced a number of initial interventions against COVID-19 by 6 March 2020 and extended these with more comprehensive interventions on 11 March (week 11) and again on 18 March. The first phase of the re-opening of society and relaxation of interventions started on 15 April (week 16). The second phase of the re-opening started on 18 May (week 20). Figure 1 shows the timeline for COVID-19 interventions in Denmark until the end of July 2020 (week 31) [4].

## Results

**Fig. 2.**
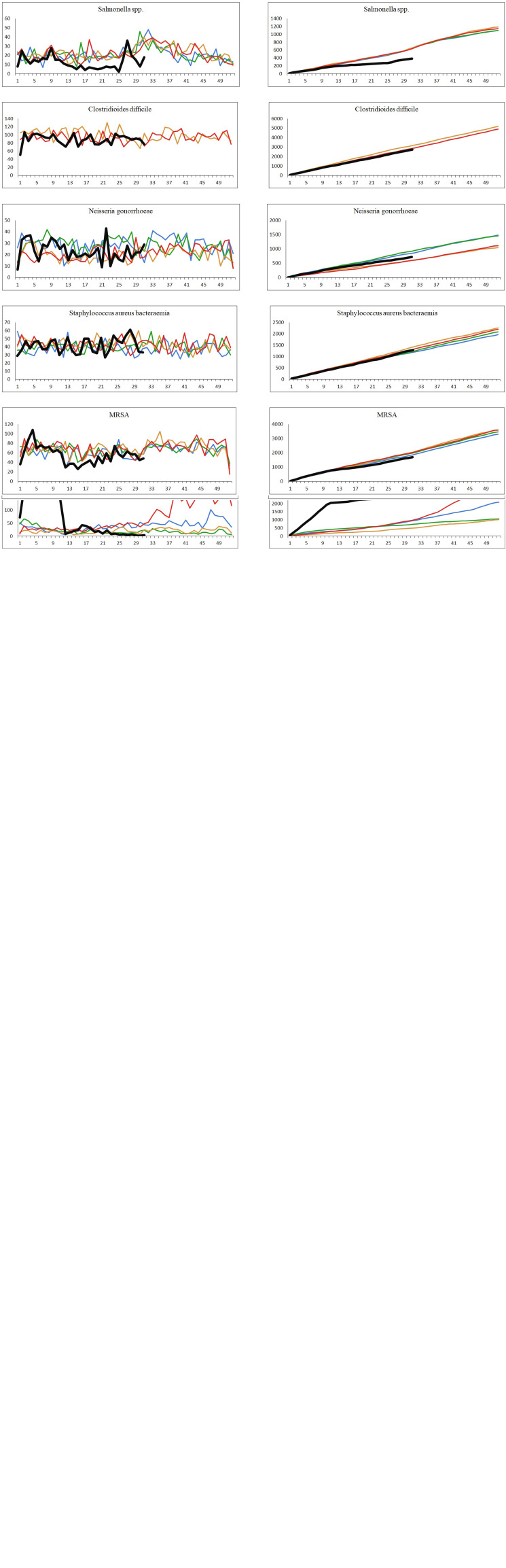
The number of cases per week and the cumulative number of cases for each year, 2016–2020. Black: year 2020; red: year 2019; orange: year 2018; green: year 2017; blue: year 2016. Left: number of confirmed cases per week. Right: cumulated number of confirmed cases per year.

The number of confirmed cases per week and the cumulative weekly number of cases for each year from 2016 to 2020 (until week 31 for year 2020) for the 16 different infectious diseases are shown in Figure 2. Due to low numbers of cases as well as similar distributions, the data for parainfluenza virus and metapneumovirus have been combined. For many of the infections studied, a decrease was observed shortly after the strict interventions were implemented in week 11. By week 14, a number of the studied infections had reached what looked like a new steady state with none or very few cases detected per week. The number of confirmed cases in the period from week 14 to 31 in 2020 compared to the same period in the years 2016–2019 are shown in Table 2.

**Table 2.** Number of registered cases in the weeks 14–31 for the year 2020 compared to the years 2016–2019

Pathogen	2016–2019 Weeks 14–31 summarised per year	2020 Weeks 14–31 summarised
Respiratory syncytial virus	110–412 (301)	69
Rhinovirus	164–501 (314)	129
Parainfluenza virus and metapneumovirus combined	169–484 (297)	0
*Mycoplasma pneumoniae*	353–1031 (573)	159
*Bordetella pertussis*	281–653 (461)	278
Invasive *Streptococcus pneumoniae*	181–221 (201)	64
Invasive *Haemophilus influenzae*	31–38 (35)	19
Invasive *Neisseria meningitidis*	11–15 (12)	4
Invasive beta-haemolytic streptococci, all	240–330 (277)	293
Invasive beta-haemolytic streptococci, group A	60–80 (71)	20
Invasive beta-haemolytic streptococci, group B, C and G	160–250 (276)	273
*Campylobacter spp.*	1532–1933 (1745)	1216
*Salmonella* spp.	382–406 (393)	188
*Clostridioides difficile*	1624–1802 (1713)	1589
*Neisseria gonorrhoeae*	322–525 (421)	378
*Staphylococcus aureus* from bacteraemia	672–830 (742)	755
Methicillin-resistant *Staphylococcus aureus*	1049–1184 (1096)	854

For 2016–2019, minimum, maximum and average for the period is shown as (min-max (average)). For *C. difficile*, the comparison period is 2018–2019.

**Fig. 3.**
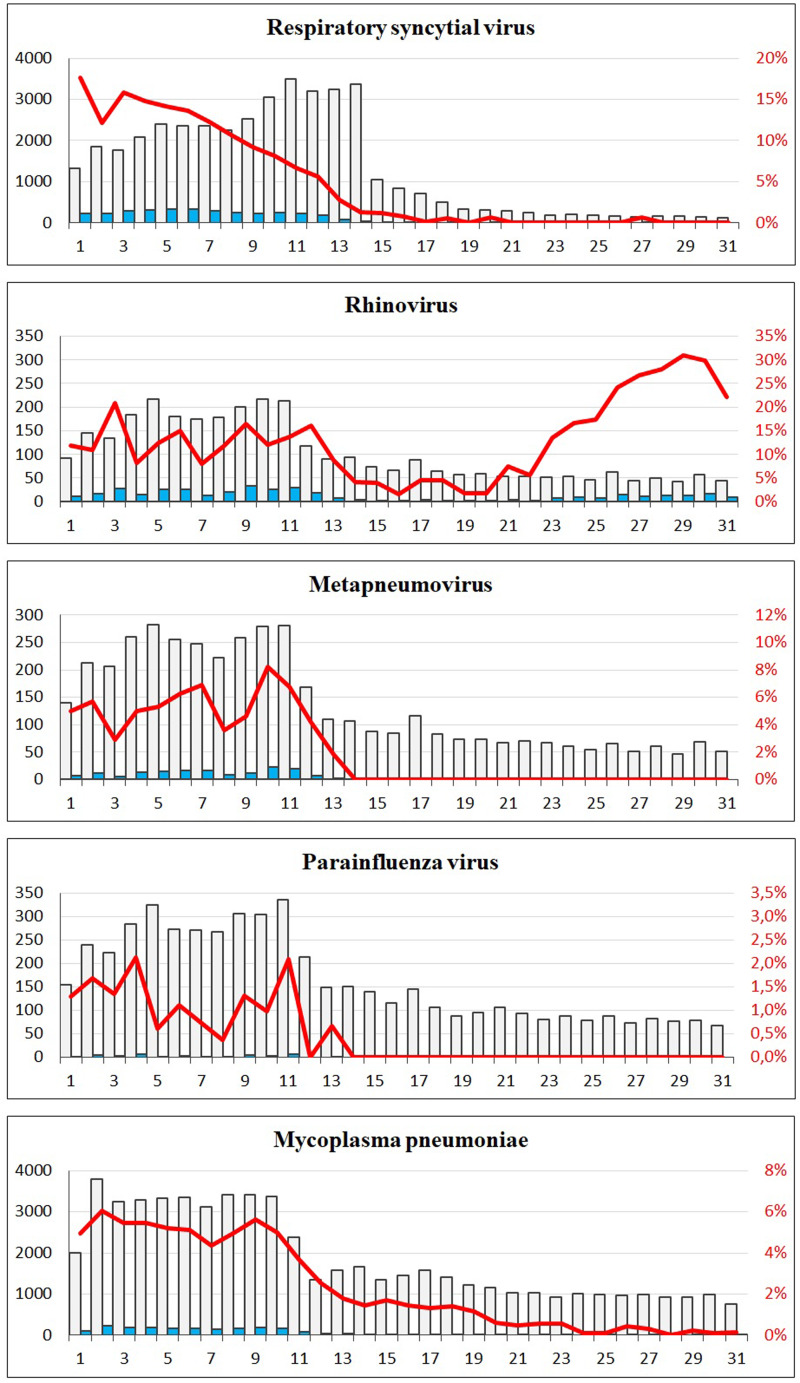
The number of tests and corresponding positive percentage for RSV, rhinovirus, metapneumovirus, parainfluenza virus and *Mycoplasma pneumoniae*, weeks 1–31, 2020. Grey bars: number of negative tests. Blue bars: number of positive tests. Red line: percentage of positive tests.

A sharp decrease in the number of confirmed cases of RSV, rhinovirus, metapneumovirus and parainfluenza virus cases was observed around week 12. For RSV, only seven confirmed cases were registered from week 17 to 31, and for metapneumovirus and parainfluenzae virus combined, the total number of cases for weeks 14–31 was zero. Later in the summer from week 21, cases of rhinovirus started to increase again and reached the levels normally seen from week 26 and onwards. For *M. pneumoniae*, only 36 cases were observed in the weeks 20–31 compared to a normal level of 29 per week on average in the previous 4 years for the same period. For all these five airway infections, a pattern of high prevalence in the winter and very low prevalence in the summer is normally observed. The COVID-19 interventions were implemented at a point in spring, where these infections are usually beginning to decline – however, the decline in 2020 was far more pronounced than what had been seen in previous years (Fig. 2). When looking at the number of diagnostic samples and corresponding percentage of positive test results for these five infections, a marked decline in the number of samples is observed very shortly after week 11. The percentage of positive tests declined as well (Fig. 3). We observed a striking decrease in confirmed cases of *B. pertussis* from week 11 staying at a low level for the remainder of the observation period. The COVID-19 interventions were implemented in the midst of an epidemic of pertussis, and the decrease from epidemic levels to very few confirmed cases is therefore extreme. The number of diagnostic analyses for pertussis decreased very dramatically from around 350 samples per day to less than 50 per day, which has recently been described in [15].

When comparing the number of confirmed cases of invasive *S. pneumoniae* with that of previous years, the decrease during spring was more pronounced in 2020 compared to previous years and remained at an unusually low level in the remainder of the observation period (Fig. 2). Regarding invasive *H. influenzae* cases, the drop in confirmed cases was less pronounced, but the cumulative number of cases showed a marked reduction in the number of cases by the end of July 2020 compared with previous years with a total of 41 cases in 2020 compared to between 65 and 78 in the previous years. During recent years, the number of confirmed invasive *N. meningitidis* cases in Denmark has been low with less than three cases per month, and differences are therefore difficult to interpret. However, for the year 2020 a complete stagnation of cases from weeks 12 to 26 was seen, and the cumulative number of cases by the end of July was 15 compared to between 19 and 33 for the previous 4 years. The decrease in cases of invasive *S. pneumoniae*, *H. influenzae* and *N. meningitidis* has recently been described briefly in an international study [10].

The number of confirmed invasive infections with beta-haemolytic streptococcci in the study period was comparable with that of previous years and neither increases nor decreases were apparent. However, a difference in the distribution of serogroups was seen with a decline in serogroup A cases (GAS). In the years 2016–2019, and in the period from 1 January to 11 March 2020, the percentage of GAS cases ranged from 23% to 29%, whereas this figure was just 8% for the period between 12 March and 31 July 2020. Only 20 cases of invasive GAS were seen in the weeks 14–31 in 2020, compared to between 60 and 80 in the corresponding period in the previous 4 years.

For the infections with foodborne transmission, the number of confirmed cases caused by *Campylobacter* spp. and *Salmonella* spp. decreased from week 11 2020. For both pathogens, the lowest number of cases per month (15 *Campylobacter* spp. and two *Salmonella* spp.) for the whole study period was seen in the period shortly after the strict interventions were initiated. However, for both pathogens, increases in incidence were later seen during the summer of 2020, and both were attributed to outbreaks [16, 17].

For cases of confirmed infections with *C. difficile*, *N. gonorrhoeae* and *S. aureus* bacteraemia, there are no seasonal patterns, and for each of these three infections, the occurrence after week 11 in 2020 was very similar to the other periods in this study. For confirmed cases of MRSA, a slight decrease in the weeks 12–21 was observed.

For the 16 infectious diseases, data on age as well as geographical region of residence for all registered cases were studied. For most of the pathogens, no change in the distribution of age was found when comparing the period from 11 March to 31 July 2020 to the full years 2016, 2017, 2018, 2019 as well as to the period from 1 January to 10 March 2020. For rhinovirus, a slight increase in the proportion of cases among children 0–9 years of age was seen at 78% (*n* = 134) of all cases compared to between 55% and 68% (mean 61%) for the other periods of the study. For pertussis, a decrease in the proportion of cases among children age 11–15 (*n* = 25) was seen at 8% of all cases compared to between 14% and 21% (mean 18%) for the other periods. For invasive infections with pneumococci, a slightly lower proportion of cases in the age group 60+ years was seen at 61% (*n* = 55) compared to between 73% and 75% (mean 74%) in the other periods of the study. For invasive infections with GAS, no cases among children age 0–14 was observed compared to a proportion of between 6% and 11% (mean 9%) in the other periods of the study – however, only 30 cases in total were registered in the period from 11 March to 31 July and, for example, 6% would therefore be less than two cases. When comparing the data for each period with respect to distribution of cases among the five Danish geographical regions, no apparent changes were observed for any of the infectious diseases studied.

## Discussion

Our nationwide population-based observational study of the impact of preventive societal measures against COVID-19 on other bacterial and viral infections showed that the interventions coincided with a marked decrease in the number of confirmed infections associated with droplet transmission. This included marked decreases in cases with confirmed RSV, rhinovirus, metapneumovirus, parainfluenza virus, *B. pertussis* and *M. pneumoniae* and also unusually low levels of cases with invasive infections caused by *S. pneumoniae*, *H. influenzae*, *N. meningitidis* and GAS. We also saw a reduction in confirmed cases associated with foodborne transmission (*Campylobacter* spp. and *Salmonella* spp.) during the lockdown. Other infectious diseases, which are not associated with droplet- or foodborne transmission (invasive BHS groups B, C and G, *C. difficile*, *N. gonorrhoeae*, *S. aureus* bacteraemia), did not show such decreases. A slight decrease in the number of confirmed cases of MRSA was however seen, and epidemiological information has confirmed that this decline was primarily attributed to fewer travel-related and community-acquired infections [18].

The lockdown in Denmark in 2020 took place during the spring where a seasonal decrease in many respiratory infections is usually seen. However, the steep decline in confirmed respiratory infections in the spring of 2020 clearly coincided with the implementation of the comprehensive lockdown from week 11 and onwards. The viral respiratory infections remained at very low levels throughout the period with COVID-19 interventions, probably in part because of a decline in the number of diagnostic tests. However, although the number of tests decreased for most of these pathogens after week 11 – the percentage of positive tests decreased simultaneously, indicating that the decreases were genuine and not a result of, for example, testing only severe cases. There was an initial recommendation during the first interventions against COVID-19 to avoid visits to the general practitioners if you had a cough, and this would also have had a considerable impact on the number of diagnostic tests made for airway infections. The number of rhinovirus cases started to increase just a few weeks after the beginning of the re-opening phase 2 where both day care centres, kindergartens and public schools had re-opened, and the proportion of cases in that period had a slightly higher than normal proportion of young children. For pertussis, the COVID-19 interventions were implemented in the midst of an epidemic, and a radical decrease in the numbers of confirmed pertussis was observed. This was however particularly due to dramatically reduced numbers of diagnostic tests. Moreover, cases of pertussis among older school children decreased in proportion during the period with interventions, and this could possibly be related to closures of schools and sports activities and the ban on large gatherings.

COVID-19 is a respiratory infection and the measures taken to reduce the risk of transmission of SARS-CoV-2 should presumably reduce the risk of other respiratory infections as well. In our study, we found a simultaneous decrease in the number of confirmed respiratory infections, a finding which is supported by other studies [6–9]. Hartnett *et al*. found that the greatest decline in emergency department visits during the early pandemic in the USA included children visits concerning respiratory infections [19]. School closures may therefore have been a contributor to the decline especially for the viral respiratory infections. This is supported by studies showing that school closures can decrease the mitigation of influenza [20] and measles [21].

We found a decrease in invasive infections caused by *S. pneumoniae*, *H. influenzae* and *N. meningitidis.* These pathogens are spread by droplet transmission similar to the other pathogens causing respiratory infections; therefore, a reduction in these infections correlating to the lockdown was expected. The observed decrease in the proportion of 60+ year olds with invasive *S. pneumoniae* was not expected, but could be related to a newly implemented recommendation for individuals 65+ years to receive a pneumococcal vaccine (PPV23). This was introduced in June 2020 [22]. We did not expect to find an impact on BHS infections or on *S. aureus* bacteraemia. These infections can be transmitted by direct contact with infected secretions, or the bacteria can be part of an individuals' microflora, but these infections are not as contagious as the viral respiratory infections, and are therefore less affected by the recommendations for physical distancing. However, we found a decline in the occurrence of invasive GAS. This is supported by the finding in a Danish surveillance report on use of antimicrobial agents and occurrence of antimicrobial resistance, where a decline in GAS, but a rise in the other BHS, was observed in Denmark during 2020 [18]. This can probably be explained by the fact that GAS is an important aetiology in pharyngo-tonsillitis in contrast to other BHS, thus making the spread of GAS more susceptible to physical distancing. There were no identified cases of invasive GAS among children age 0–14 in the weeks 14–31 in 2020, but the total number of cases was generally low for this period. We did not expect a decline in testing regarding invasive infections, since the seriousness of these infections would presumably have resulted in contact to the healthcare system for almost all cases.

For the two foodborne infectious diseases studied (*Campylobacter* spp. and *Salmonella* spp.), decreases in the number of confirmed cases were seen, and this was to a large extend linked to the travel restrictions imposed, but possible also caused by fewer social gatherings and the closures of restaurants, cafés and workplace canteens [23]. A decrease in the number of diagnostic tests cannot be ruled out. The spike in the incidence of *Campylobacter* spp. in week 23 2020 was due to a local outbreak, where more than 100 individuals were infected [16]. The spike in the incidence of *Salmonella* spp. in week 27 was due to coinciding outbreaks of *Salmonella* Strathcona and *Salmonella* Kasenyi [17, 23].

For four of the five infectious diseases not related to airway or foodborne transmission (invasive BHS groups B/C/G, *C. difficile*, *N. gonorrhoeae* and *S. aureus* bacteraemia), no apparent effect of the interventions was seen. We found no change in *C. difficile* infections in the beginning of the lockdown when compared to the years 2018 and 2019. *C. difficile* is transmitted by person to person contact, with antibiotic therapy, old age and hospital stay being central risk factors [24]. During the lockdown, many non-acute surgeries were postponed to free hospital capacity for COVID-19 patients. Consequently, we expected a slight decrease in *C. difficile* infections correlating to a lower number of hospital stays. However, there was a number of individuals with older age or comorbidities admitted to intensive care units in the spring 2020 due to COVID-19 and such patients often receive antibiotics. This could potentially balance out the effect of a reduced number of elective surgeries. Alternatively, the postponed elective surgeries could possibly have been mainly in healthy adults and not in older individuals with comorbidities.

The decrease found in MRSA registrations correlating to the lockdown could be a result of less travel or due to physical distancing. However, since most MRSA registrations are due to minor infections or screening for carriage, the observed decrease could also be caused by less testing and/or less contact to general practitioners during the lockdown. Data on number of tests are not available.

There were some limitations to our study. Most importantly, we only have data on confirmed cases and the true burden of disease is therefore unknown. This is however true for the whole study period, and not just for the period with COVID-19 interventions in place. For some of the pathogens, we did not have data on the number of tests performed; therefore, we cannot rule out that a decrease in the number of registered infections was due to less testing because of the lockdown. However, less testing would likely only have had a minor impact on the invasive infections, since we assume that the majority of patients with suspicions of invasive bacterial infections would have a test performed regardless of the lockdown. Different behaviour in when to seek medical attention for less severe symptoms, and different routines at general practitioners and hospitals will likely also have had an impact on the number of diagnostic analyses for some of the infections studied. A fear of contracting COVID-19 when visiting medical professionals might also have had an effect on the number of diagnostic samples taken. As yet unknown interactions between the different infectious diseases studied could perhaps also have had an effect on the observed patterns. We did not have access to clinical data about the severity of the diseases and therefore cannot consider this. We found no geographical difference in the distribution of confirmed cases, and the behaviour of the population in the five different regions of Denmark can therefore be assumed to have been comparable. We have looked at the period with COVID-19 interventions as a whole, and not at each of the phases or interventions. Further studies are needed to delineate the impact of the individual interventions.

In conclusion, we find that the COVID-19-related widespread non-pharmaceutical interventions such as physical distancing, less travel and a lockdown of schools, restaurants and workplaces coincided with a major decline in respiratory infections, invasive infections related to airway transmission and, to a smaller extent, some foodborne-transmitted infections. The effects, if any, of each of these interventions can however not be isolated. Additional changes in behaviour among the population due to fear of becoming infected, as well as changes in routines for diagnostic testing at health care facilities are also likely to have had an impact on the observed decreases. Although such drastic interventions should not be introduced in a non-pandemic situation, it is reassuringly suggested from our findings, that non-pharmaceutical interventions work very effectively and can have a substantial impact on the wider burden of infectious diseases.

## Data Availability

The data that support the findings of this study are available from the corresponding author, TD, upon reasonable request.
